# Supplementary material for: The effect of molnupiravir and nirmatrelvir on SARS-CoV-2 genome diversity in severe models of COVID-19
Source: bioRxiv. 2024 Dec 20:2024.02.27.582110. Originally published 2024 Feb 28. Preprint. [Version 3] doi: 10.1101/2024.02.27.582110 (PMC10925244; doi:10.1101/2024.02.27.582110)
Supplement: Supplement 2 [file NIHPP2024.02.27.582110v3-supplement-2.pdf]

bioRxiv preprint doi: https://doi.org/10.1101/2024.02.27.582110; this version posted December 20, 2024. The copyright holder for this preprint (which was not certified by peer review) is the author/funder, who has granted bioRxiv a license to display the preprint in perpetuity. It is made available under aCC-BY 4.0 International license.

SUPPLEMENTARY TABLES

Table S1: *The number of samples per cohort, sample type and DPI that were included in the sequencing analysis. Samples with less than 90% coverage and poor or mediocre quality control results determined by Nextclade CLI, were excluded from the analysis. *Multiple end point time-points due to humane end-point variation.*

Cohort	Sample type	DPI*	*n*
**Cyclophosphamide and Molnupiravir**	Swab	1	4
		3	4
		5	3
	Lung Tissue	7	4
	Nasal Tissue	7	4
**Cyclophosphamide and Nirmatrelvir**	Swab	1	1
		3	2
		5	1
	Lung Tissue	4	1
		7	2
	Nasal Tissue	4	1
		7	2
**Cyclophosphamide only**	Swab	1	4
		3	4
		4	1
		5	2
	Lung Tissue	4	2
		6	2
	Nasal Tissue	4	1
		6	2
**Cyclophosphamide, Nirmatrelvir and Molnupiravir**	Swab	1	1
		3	2

bioRxiv preprint doi: https://doi.org/10.1101/2024.02.27.582110; this version posted December 20, 2024. The copyright holder for this preprint (which was not certified by peer review) is the author/funder, who has granted bioRxiv a license to display the preprint in perpetuity. It is made available under aCC-BY 4.0 International license.

**Cohort**	**Sample type**	**DPI***	***n***
	Lung Tissue	3	1
		7	2
	Nasal Tissue	3	1
		7	3
**Molnupiravir**	Swab	1	4
		3	4
		5	1
	Lung Tissue	7	3
	Nasal Tissue	7	1
**Vehicle**	Swab	1	4
		3	4
		5	4
	Lung Tissue	6	4
	Nasal Tissue	6	3

Table S2: *Unique phenotypic changes in SARS-CoV-2 within the dataset. Input virus amino acids and S: H665Y were excluded.*

**Amino acid change**	**Occurrence in dataset**	**Tissue type**	**Cohorts**
**S: D215H**	2	Swab	Vehicle, Cyclophosphamide, Molnupiravir &

bioRxiv preprint doi: https://doi.org/10.1101/2024.02.27.582110; this version posted December 20, 2024. The copyright holder for this preprint (which was not certified by peer review) is the author/funder, who has granted bioRxiv a license to display the preprint in perpetuity. It is made available under aCC-BY 4.0 International license.

Amino acid change	Occurrence in dataset	Tissue type	Cohorts
			nirmatrelvir
**ORF1a: G2581S**	1	Swab	Cyclophosphamide & nirmatrelvir
**ORF3a: P42L**	1	Swab	Cyclophosphamide & Molnupiravir
**ORF3a: Q57H**	2	Nasal Tissue<br>Swab	Cyclophosphamide, Molnupiravir & nirmatrelvir,<br>Cyclophosphamide only
**E: Y42H**	1	Swab	Cyclophosphamide & nirmatrelvir
**ORF1a: G82D**	1	Lung Tissue	Cyclophosphamide, nirmatrelvir and Molnupiravir
**S: V47I**			Cyclophosphamide, nirmatrelvir and Molnupiravir
**ORF1a: T568I**	1	Nasal Tissue	Cyclophosphamide, nirmatrelvir and Molnupiravir
**ORF1a: C2160Y**			
**ORF1b: F312L**			
**ORF1b: V2515I**			
**ORF8: F108L**			
**S: A27T**			
**ORF1a: F741L**	1	Nasal Tissue	Cyclophosphamide & nirmatrelvir
**ORF1a: A872T**			
**ORF7b: S5P**			
**S: A890V**			
**S: D985N**			

bioRxiv preprint doi: https://doi.org/10.1101/2024.02.27.582110; this version posted December 20, 2024. The copyright holder for this preprint (which was not certified by peer review) is the author/funder, who has granted bioRxiv a license to display the preprint in perpetuity. It is made available under aCC-BY 4.0 International license.

Amino acid change	Occurrence in dataset	Tissue type	Cohorts
**ORF1a: F190L**	1	Nasal Tissue	Molnupiravir
**ORF1a: G209S**			
**ORF1a: V2130I**			
**ORF1b: R1729H**			
**ORF3a:M1I**			
**ORF3a: G224S**			
**S: S247R**			
**S: V483I**			

bioRxiv preprint doi: https://doi.org/10.1101/2024.02.27.582110; this version posted December 20, 2024. The copyright holder for this preprint (which was not certified by peer review) is the author/funder, who has granted bioRxiv a license to display the preprint in perpetuity. It is made available under aCC-BY 4.0 International license.

Supplementary Figures

Figure S1

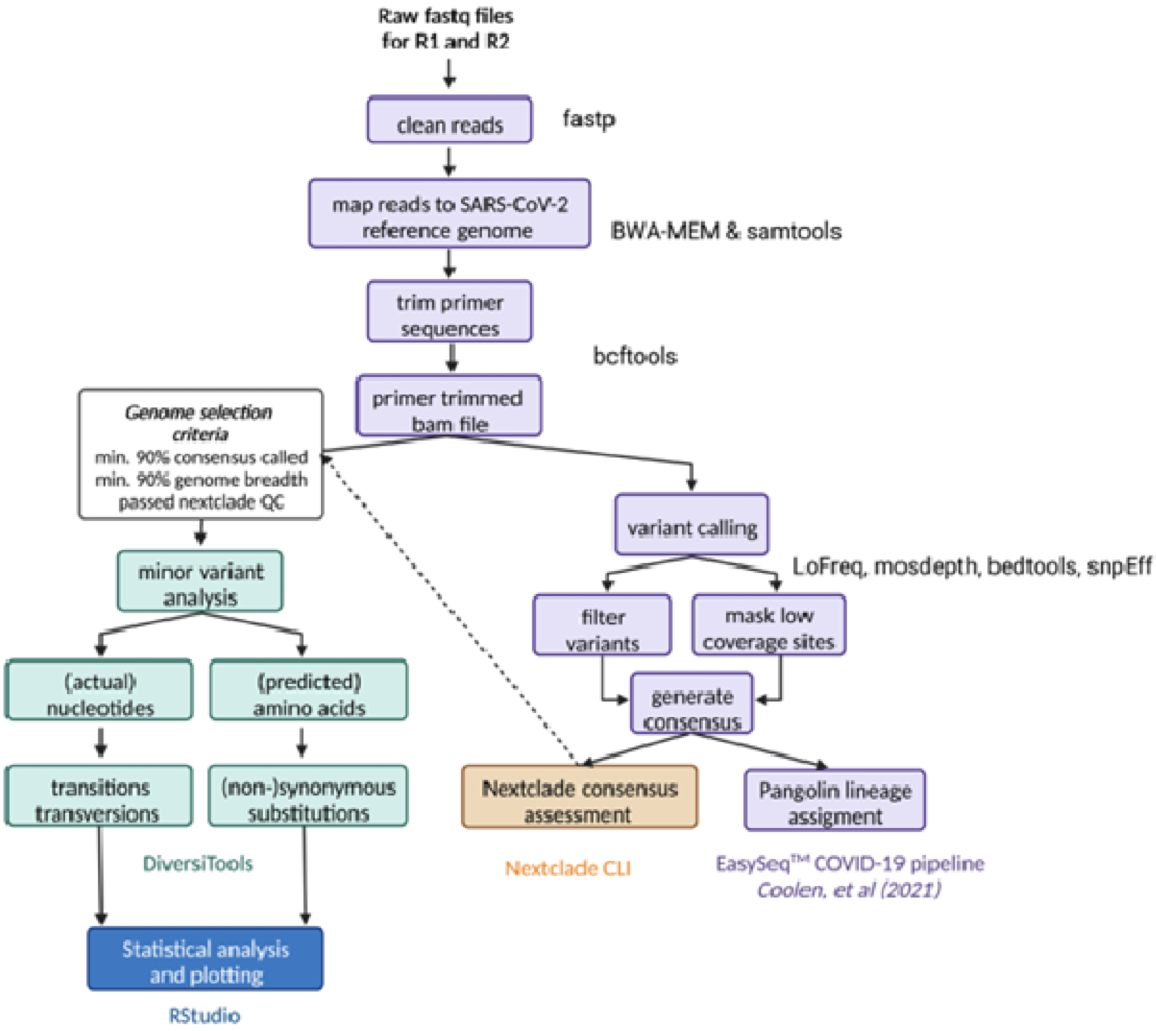

Fig S1: Bioinformatic workflow used for sequencing analysis.

bioRxiv preprint doi: https://doi.org/10.1101/2024.02.27.582110; this version posted December 20, 2024. The copyright holder for this preprint (which was not certified by peer review) is the author/funder, who has granted bioRxiv a license to display the preprint in perpetuity. It is made available under aCC-BY 4.0 International license.

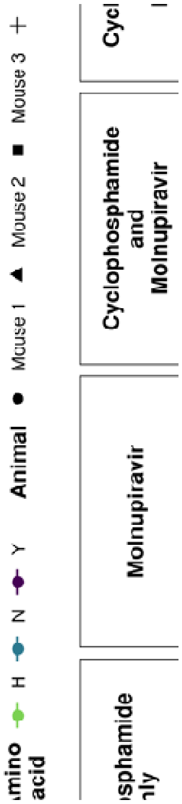

Fig S2: The proportion of amino acid H and Y at position 655 in the Spike protein over time or in lung or nasal tissues. The S: H655Y mutation was observed at the minor variant level if not observed in the consensus level. Each point represents one animal within the cohort.

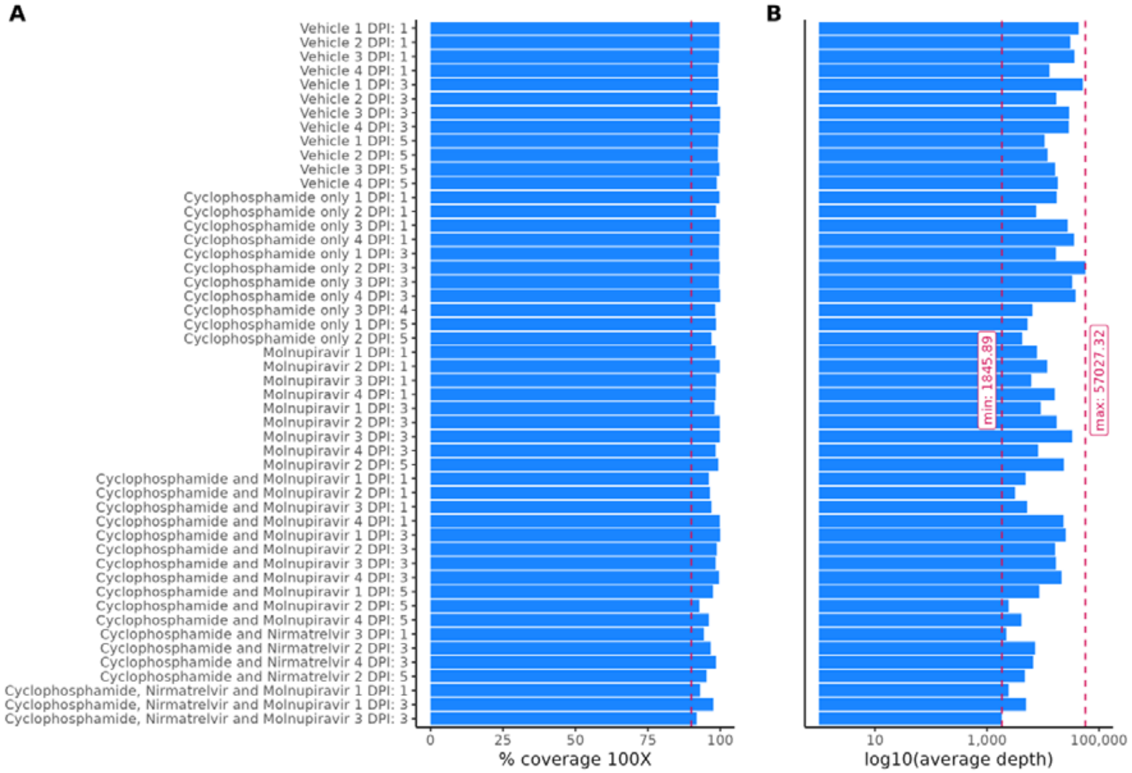

Fig S3: The coverage (A) and average depth (B) of the swabs used in the minor variant analysis.

bioRxiv preprint doi: https://doi.org/10.1101/2024.02.27.582110; this version posted December 20, 2024. The copyright holder for this preprint (which was not certified by peer review) is the author/funder, who has granted bioRxiv a license to display the preprint in perpetuity. It is made available under aCC-BY 4.0 International license.

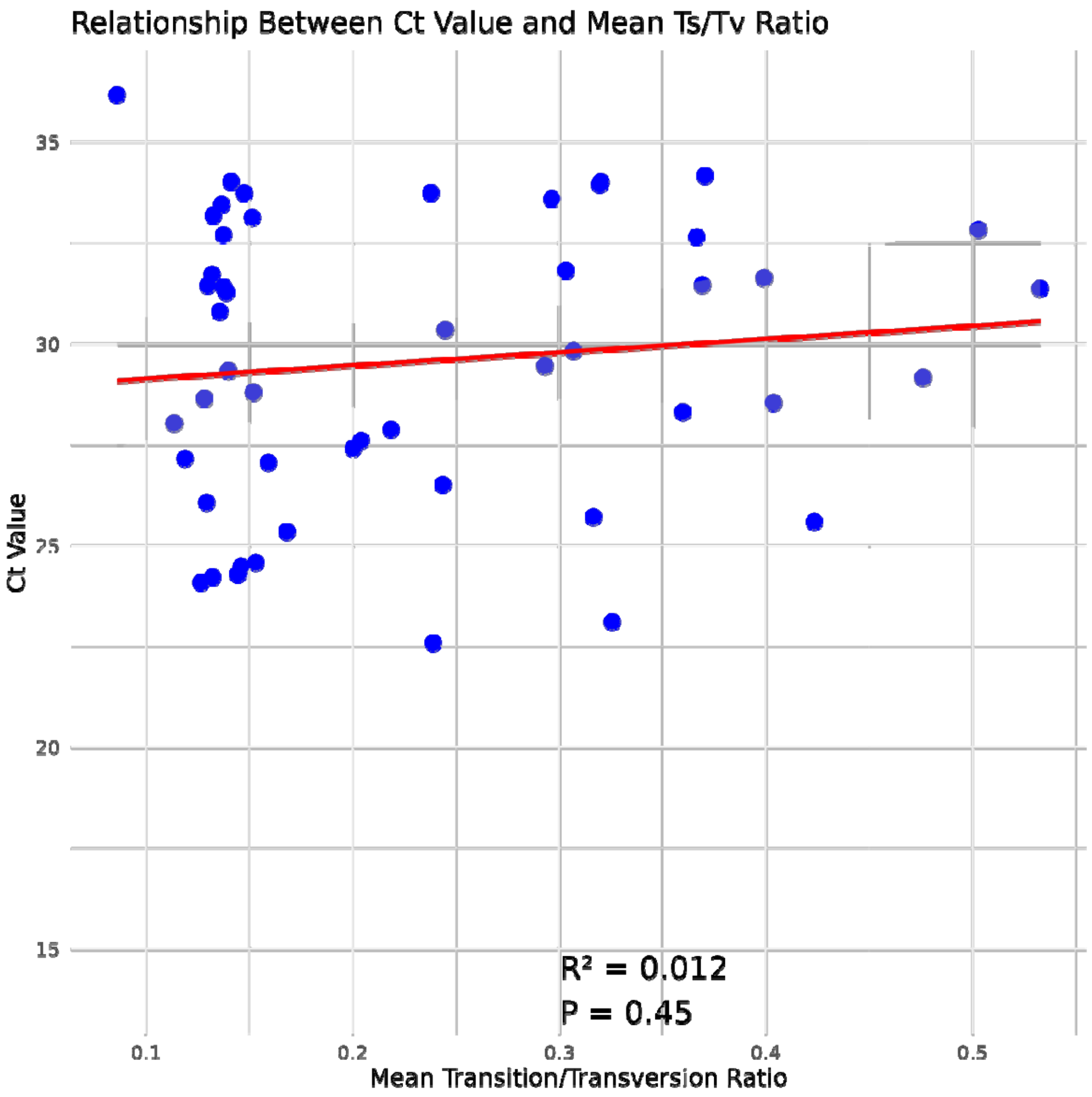

Fig S4: The scatter plot shows the relationship between Ct values and the mean transition/transversion (Ts/Tv) ratios calculated with the DiversiTools output. Each blue dot represents a swab sample used in the minor variant analysis. The red line indicates the linear regression fit, with the shaded region showing the 95% confidence interval. Statistical analysis revealed no significant correlation between Ct values and Ts/Tv ratios (R^2 = 0.012 , p = 0.45), supporting the conclusion that there is no association between the two variables.

bioRxiv preprint doi: https://doi.org/10.1101/2024.02.27.582110; this version posted December 20, 2024. The copyright holder for this preprint (which was not certified by peer review) is the author/funder, who has granted bioRxiv a license to display the preprint in perpetuity. It is made available under aCC-BY 4.0 International license.

bioRxiv preprint doi: https://doi.org/10.1101/2024.02.27.582110; this version posted December 20, 2024. The copyright holder for this preprint (which was not certified by peer review) is the author/funder, who has granted bioRxiv a license to display the preprint in perpetuity. It is made available under aCC-BY 4.0 International license.

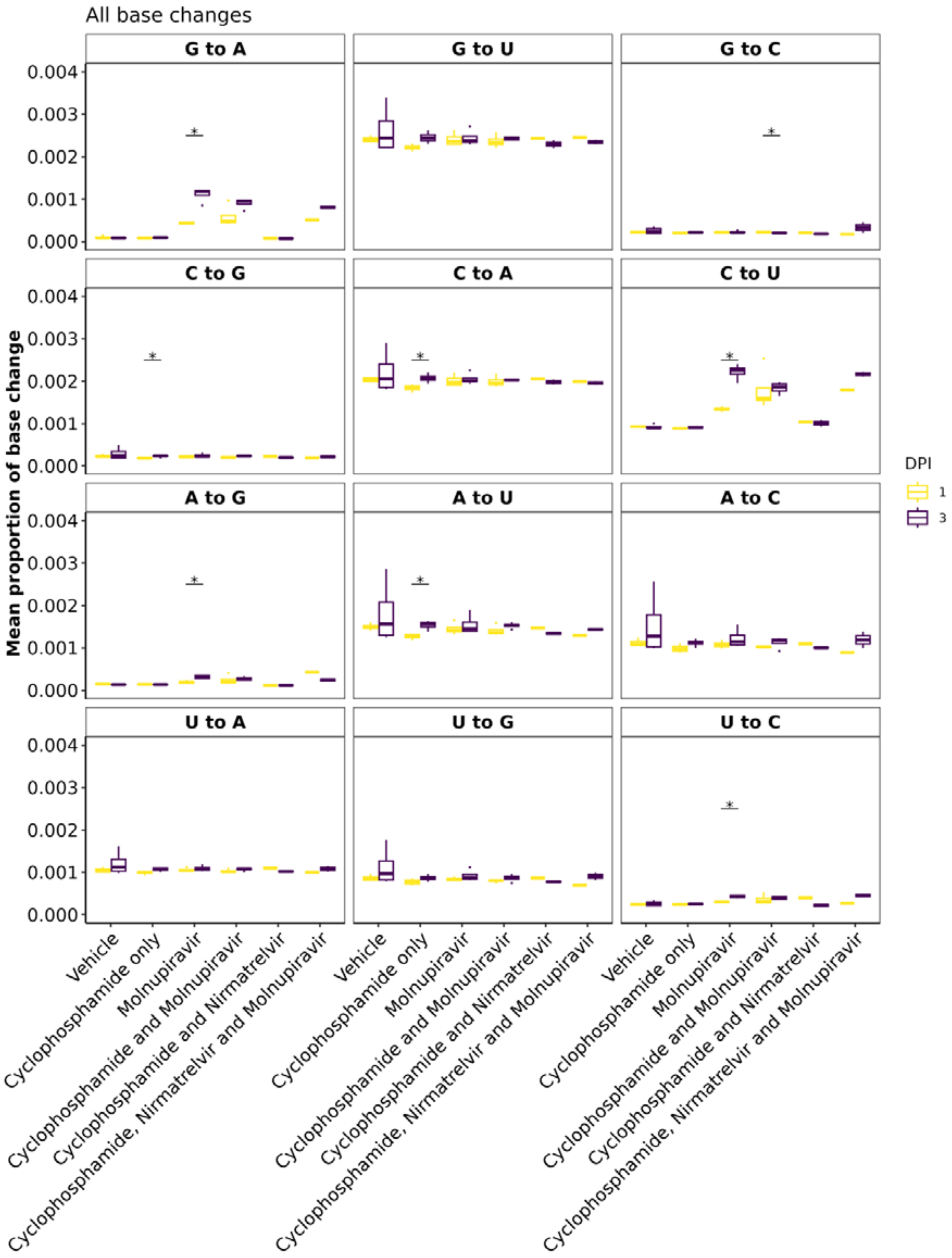

*Fig S5: Minor variation for all base changes between day 1 and day 3 post infection. * represents a P value <0.05 (Mann Whitney U test).*
