# Supplementary material for: The effect of molnupiravir and nirmatrelvir on SARS-CoV-2 genome diversity in severe models of COVID-19
Source: bioRxiv. 2024 Jun 14:2024.02.27.582110. Originally published 2024 Feb 28. Preprint. [Version 2] doi: 10.1101/2024.02.27.582110 (PMC10925244; doi:10.1101/2024.02.27.582110)
Supplement: Supplementary file 1 [file NIHPP2024.02.27.582110V2-supplement-1.pdf]

bioRxiv preprint doi: https://doi.org/10.1101/2024.02.27.582110; this version posted June 14, 2024. The copyright holder for this preprint (which was not certified by peer review) is the author/funder, who has granted bioRxiv a license to display the preprint in perpetuity. It is made available under aCC-BY 4.0 International license.

# SUPPLEMENTARY TABLES

***Table S1:*** *The number of samples per cohort, sample type and DPI that were included in the sequencing analysis. Samples with less than 90% coverage and poor or mediocre quality control results determined by Nextclade CLI, were excluded from the analysis. *Multiple end point time-points due to humane end-point variation.*

| Cohort | Sample type | DPI* | *n* |
|---|---|---|---|
| **Cyclophosphamide and Molnupiravir** | Swab | 1 | 4 |
| | | 3 | 4 |
| | | 5 | 3 |
| | Lung Tissue | 7 | 4 |
| | Nasal Tissue | 7 | 4 |
| **Cyclophosphamide and nirmatrelvir** | Swab | 1 | 1 |
| | | 3 | 2 |
| | | 5 | 1 |
| | Lung Tissue | 4 | 1 |
| | | 7 | 2 |
| | Nasal Tissue | 4 | 1 |
| | | 7 | 2 |
| **Cyclophosphamide only** | Swab | 1 | 4 |
| | | 3 | 4 |
| | | 4 | 1 |
| | | 5 | 2 |
| | Lung Tissue | 4 | 2 |
| | | 6 | 2 |
| | Nasal Tissue | 4 | 1 |
| | | 6 | 2 |
| **Cyclophosphamide, nirmatrelvir and Molnupiravir** | Swab | 1 | 1 |
| | | 3 | 2 |

bioRxiv preprint doi: https://doi.org/10.1101/2024.02.27.582110; this version posted June 14, 2024. The copyright holder for this preprint (which was not certified by peer review) is the author/funder, who has granted bioRxiv a license to display the preprint in perpetuity. It is made available under aCC-BY 4.0 International license.

| **Cohort** | **Sample type** | **DPI*** | ***n*** |
|---|---|---|---|
| | Lung Tissue | 3 | 1 |
| | | 7 | 2 |
| | Nasal Tissue | 3 | 1 |
| | | 7 | 3 |
| **Molnupiravir** | Swab | 1 | 4 |
| | | 3 | 4 |
| | | 5 | 1 |
| | Lung Tissue | 7 | 3 |
| | Nasal Tissue | 7 | 1 |
| **Vehicle** | Swab | 1 | 4 |
| | | 3 | 4 |
| | | 5 | 4 |
| | Lung Tissue | 6 | 4 |
| | Nasal Tissue | 6 | 3 |

## Table S2: Input virus

| **Input virus** | | |
|---|---|---|
| **Nucleotide change** | **Gene** | **Amino Acid Change** |
| A6948C | Nsp3 | N1410T or N2228T |
| G11083T | Nsp6 | L37F or L3606F |
| C21005T | Nsp16 | A116V or A2513V |
| C25452T | Orf3a | I20 no change |
| C28253T | Orf8 | F120 no change |

***Table S3:*** *Unique phenotypic changes in SARS-CoV-2 within the dataset. Input virus amino acids and S: H665Y were excluded.*

bioRxiv preprint doi: https://doi.org/10.1101/2024.02.27.582110; this version posted June 14, 2024. The copyright holder for this preprint (which was not certified by peer review) is the author/funder, who has granted bioRxiv a license to display the preprint in perpetuity. It is made available under aCC-BY 4.0 International license.

| Amino acid change | Occurrence in dataset | Tissue type | Cohorts |
|---|---|---|---|
| **S: D215H** | 2 | Swab | Vehicle, Cyclophosphamide, Molnupiravir & nirmatrelvir |
| **ORF1a: G2581S** | 1 | Swab | Cyclophosphamide & nirmatrelvir |
| **ORF3a: P42L** | 1 | Swab | Cyclophosphamide & Molnupiravir |
| **ORF3a: Q57H** | 2 | Nasal Tissue Swab | Cyclophosphamide, Molnupiravir & nirmatrelvir, Cyclophosphamide only |
| **E: Y42H** | 1 | Swab | Cyclophosphamide & nirmatrelvir |
| **ORF1a: G82D** | 1 | Lung Tissue | Cyclophosphamide, nirmatrelvir and Molnupiravir Cyclophosphamide, nirmatrelvir and Molnupiravir |
| **S: V47I** | | | |
| **ORF1a: T568I** | 1 | Nasal Tissue | Cyclophosphamide, nirmatrelvir and Molnupiravir |
| **ORF1a: C2160Y** | | | |
| **ORF1b: F312L** | | | |
| **ORF1b: V2515I** | | | |
| **ORF8: F108L** | | | |
| **S: A27T** | | | |
| **ORF1a: F741L** | 1 | Nasal Tissue | Cyclophosphamide & nirmatrelvir |
| **ORF1a: A872T** | | | |
| **ORF7b: S5P** | | | |

bioRxiv preprint doi: https://doi.org/10.1101/2024.02.27.582110; this version posted June 14, 2024. The copyright holder for this preprint (which was not certified by peer review) is the author/funder, who has granted bioRxiv a license to display the preprint in perpetuity. It is made available under aCC-BY 4.0 International license.

| Amino acid change | Occurrence in dataset | Tissue type | Cohorts |
|---|---|---|---|
| **S: A890V** | | | |
| **S: D985N** | | | |
| **ORF1a: F190L** | 1 | Nasal Tissue | Molnupiravir |
| **ORF1a: G209S** | | | |
| **ORF1a: V2130I** | | | |
| **ORF1b: R1729H** | | | |
| **ORF3a:M1I** | | | |
| **ORF3a: G224S** | | | |
| **S: S247R** | | | |
| **S: V483I** | | | |

bioRxiv preprint doi: https://doi.org/10.1101/2024.02.27.582110; this version posted June 14, 2024. The copyright holder for this preprint (which was not certified by peer review) is the author/funder, who has granted bioRxiv a license to display the preprint in perpetuity. It is made available under aCC-BY 4.0 International license.

# Supplementary Figures

## Figure S1

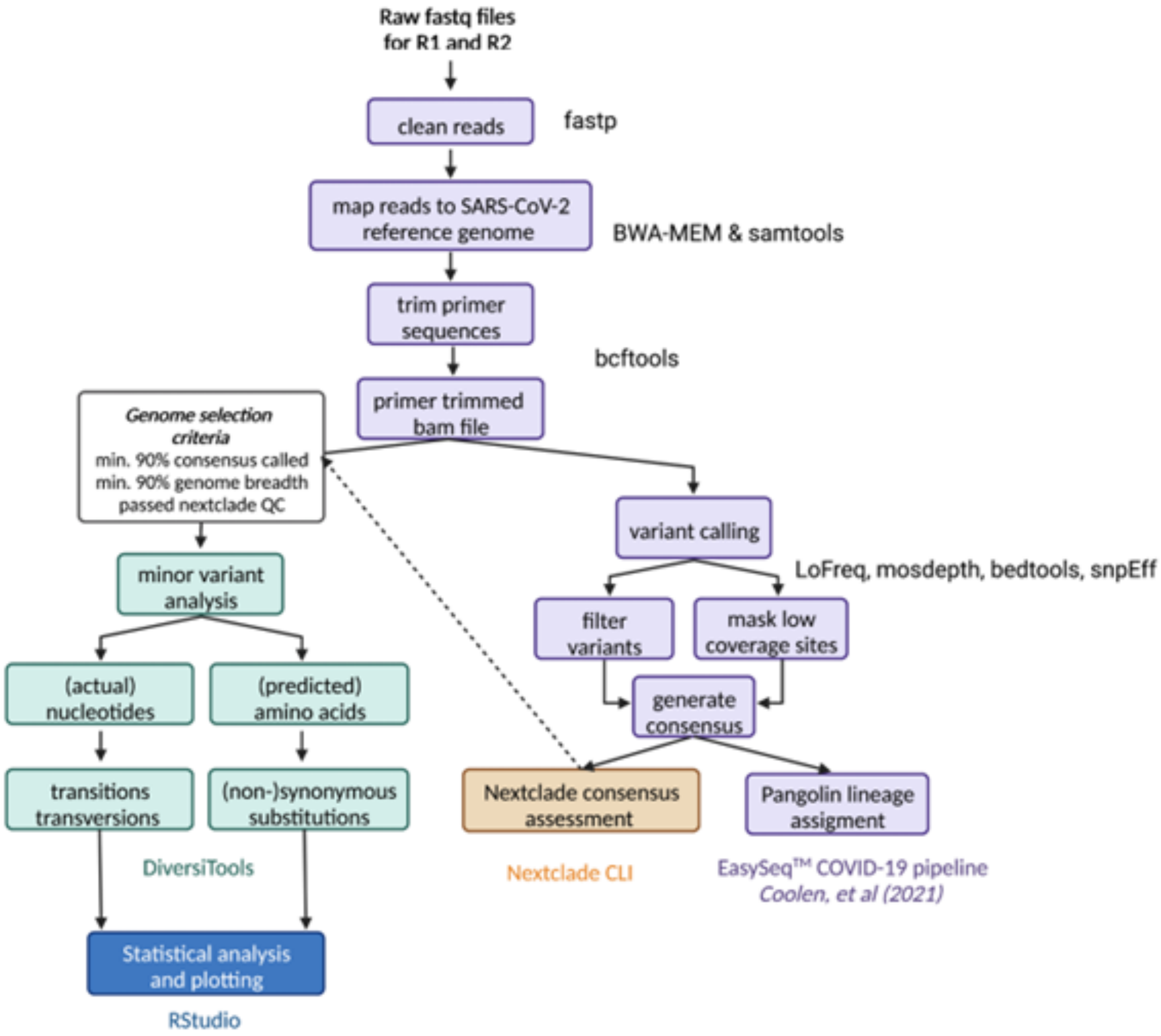


Bioinformatic workflow used for sequencing analysis.

**Figure S2**

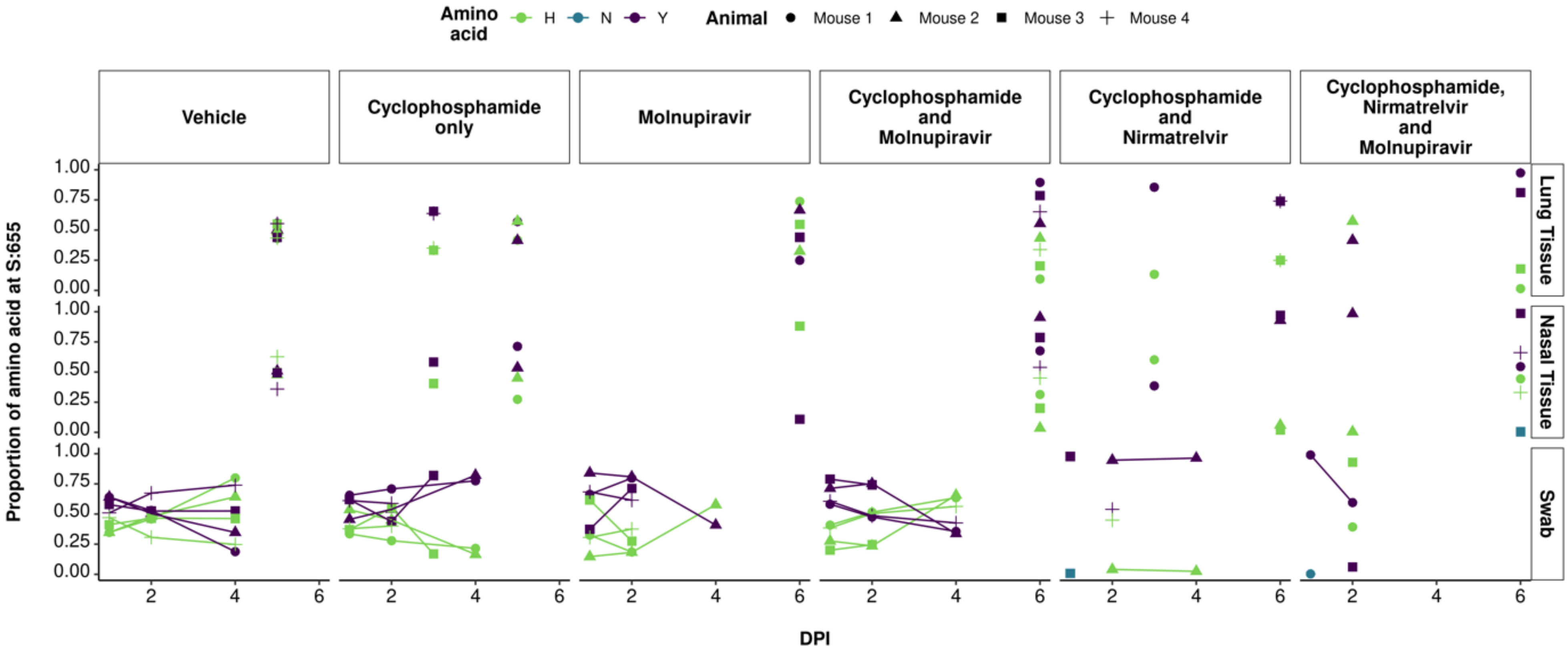


The proportion of amino acid H and Y at position 655 in the Spike protein over time or in lung or nasal tissues. The S: H655Y mutation was observed at the minor variant level if not observed in the consensus level. Each point represents one animal within the cohort.
