# Supplementary material for: The effect of molnupiravir and nirmatrelvir on SARS-CoV-2 genome diversity in infected and immune suppressed mice
Source: bioRxiv. 2024 Feb 28:2024.02.27.582110. Preprint. [Version 1] doi: 10.1101/2024.02.27.582110 (PMC10925244; doi:10.1101/2024.02.27.582110)
Supplement: Supplementary file 1 [file media-1.pdf]

SUPPLEMENTARY TABLES

Table S1: *The number of samples per cohort, sample type and DPI that were included in the sequencing analysis. Samples with less than 90% coverage and poor or mediocre quality control results determined by Nextclade CLI, were excluded from the analysis. *Multiple end point time-points due to humane end-point variation.*

Cohort	Sample type	DPI*	*n*
**Cyclophosphamide and Molnupiravir**	Swab	1	4
		3	4
		5	3
	Lung Tissue	7	4
	Nasal Tissue	7	4
**Cyclophosphamide and nirmatrelvir**	Swab	1	1
		3	2
		5	1
	Lung Tissue	4	1
		7	2
	Nasal Tissue	4	1
		7	2
**Cyclophosphamide only**	Swab	1	4
		3	4
		4	1
		5	2
	Lung Tissue	4	2
		6	2
	Nasal Tissue	4	1
		6	2
	Swab	1	1

Cohort	Sample type	DPI*	*n*
**Cyclophosphamide, nirmatrelvir and Molnupiravir**		3	2
	Lung Tissue	3	1
		7	2
	Nasal Tissue	3	1
		7	3
**Molnupiravir**	Swab	1	4
		3	4
		5	1
	Lung Tissue	7	3
	Nasal Tissue	7	1
**Vehicle**	Swab	1	4
		3	4
		5	4
	Lung Tissue	6	4
	Nasal Tissue	6	3

Table S2: Input virus

**Input virus**		
**Nucleotide change**	**Gene**	**Amino Acid Change**
A6948C	Nsp3	N1410T or N2228T
G11083T	Nsp6	L37F or L3606F
C21005T	Nsp16	A116V or A2513V
C25452T	Orf3a	I20 no change
C28253T	Orf8	F120 no change

Table S3: *Unique phenotypic changes in SARS-CoV-2 within the dataset. Input virus amino acids and S: H665Y were excluded.*

Amino acid change	Occurrence in dataset	Tissue type	Cohorts
**S: D215H**	2	Swab	Vehicle, Cyclophosphamide, Molnupiravir & nirmatrelvir
**ORF1a: G2581S**	1	Swab	Cyclophosphamide & nirmatrelvir
**ORF3a: P42L**	1	Swab	Cyclophosphamide & Molnupiravir
**ORF3a: Q57H**	2	Nasal Tissue<br>Swab	Cyclophosphamide, Molnupiravir & nirmatrelvir, Cyclophosphamide only
**E: Y42H**	1	Swab	Cyclophosphamide & nirmatrelvir
**ORF1a: G82D**	1	Lung Tissue	Cyclophosphamide, nirmatrelvir and Molnupiravir
**S: V47I**			Cyclophosphamide, nirmatrelvir and Molnupiravir
**ORF1a: T568I**	1	Nasal Tissue	Cyclophosphamide, nirmatrelvir and Molnupiravir
**ORF1a: C2160Y**			
**ORF1b: F312L**			
**ORF1b: V2515I**			
**ORF8: F108L**			
**S: A27T**			
**ORF1a: F741L**	1	Nasal Tissue	

Amino acid change	Occurrence in dataset	Tissue type	Cohorts
**ORF1a: A872T**			Cyclophosphamide & nirmatrelvir
**ORF7b: S5P**			
**S: A890V**			
**S: D985N**			
**ORF1a: F190L**	1	Nasal Tissue	Molnupiravir
**ORF1a: G209S**			
**ORF1a: V2130I**			
**ORF1b: R1729H**			
**ORF3a:M1I**			
**ORF3a: G224S**			
**S: S247R**			
**S: V483I**			

Supplementary Figures

Figure S1

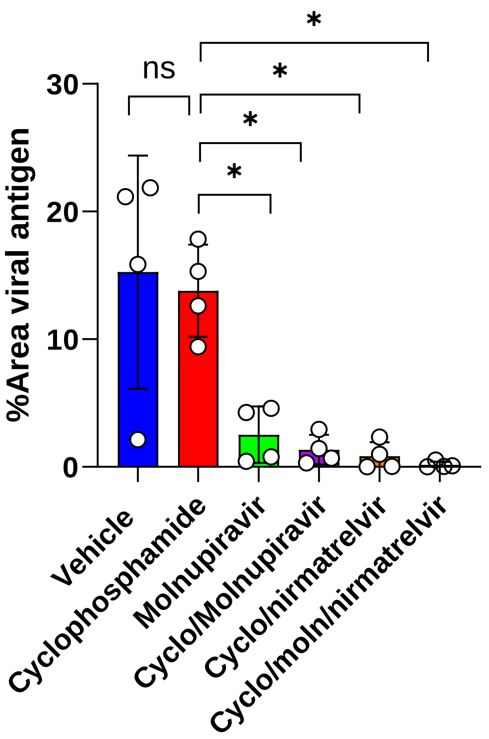

Morphometric analysis of scanned sections stained by immunohistology for the expression of SARS-CoV-2 nucleoprotein was performed using the software programme Visiopharm to quantify the area stained for viral antigen. Points represent individual animals with bar at the mean. Pairwise comparisons were made between groups using a Mann-Whitney U test. * represents $P < 0.05$.

Figure S2

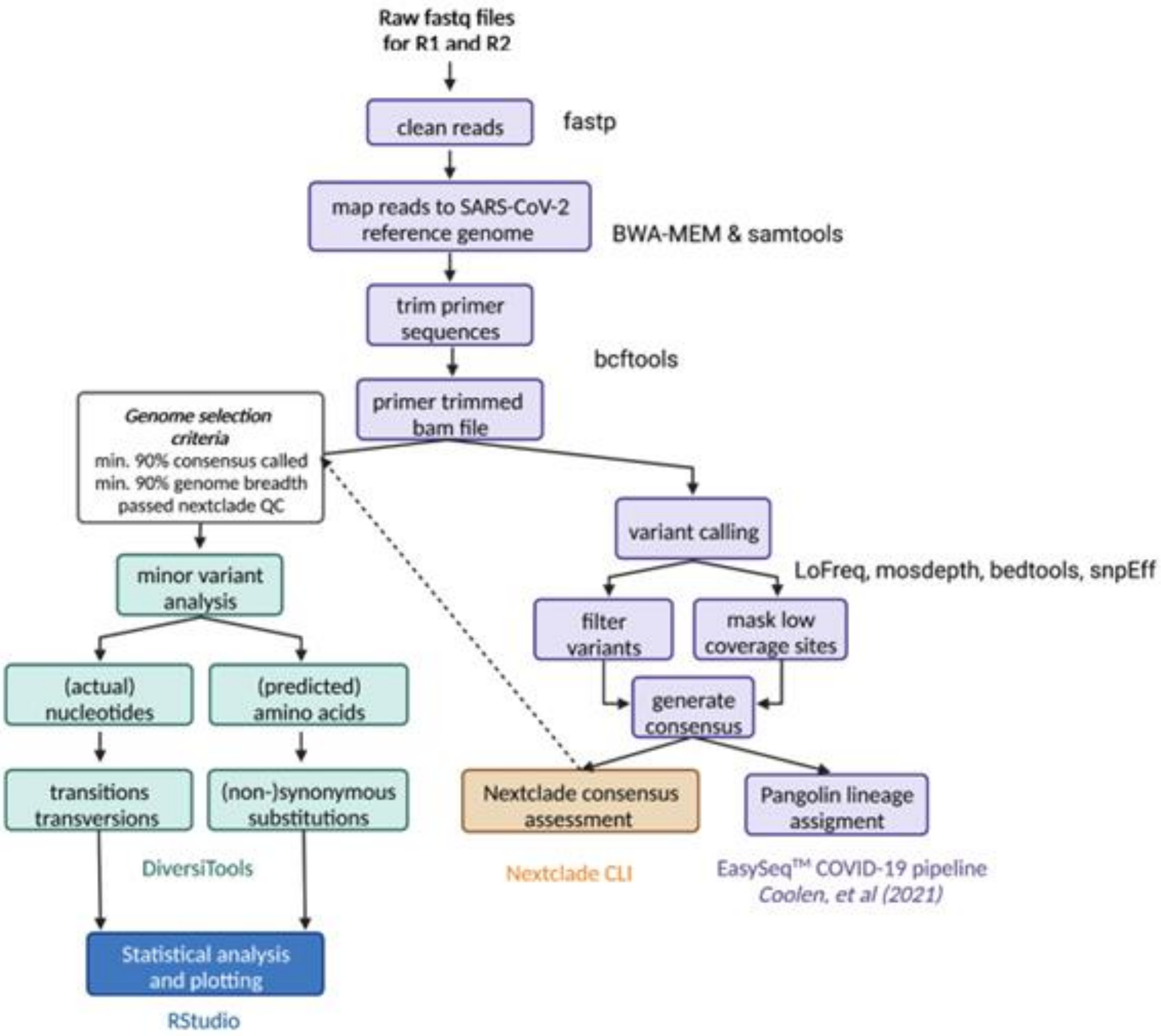

Bioinformatic workflow used for sequencing analysis.

Figure S3

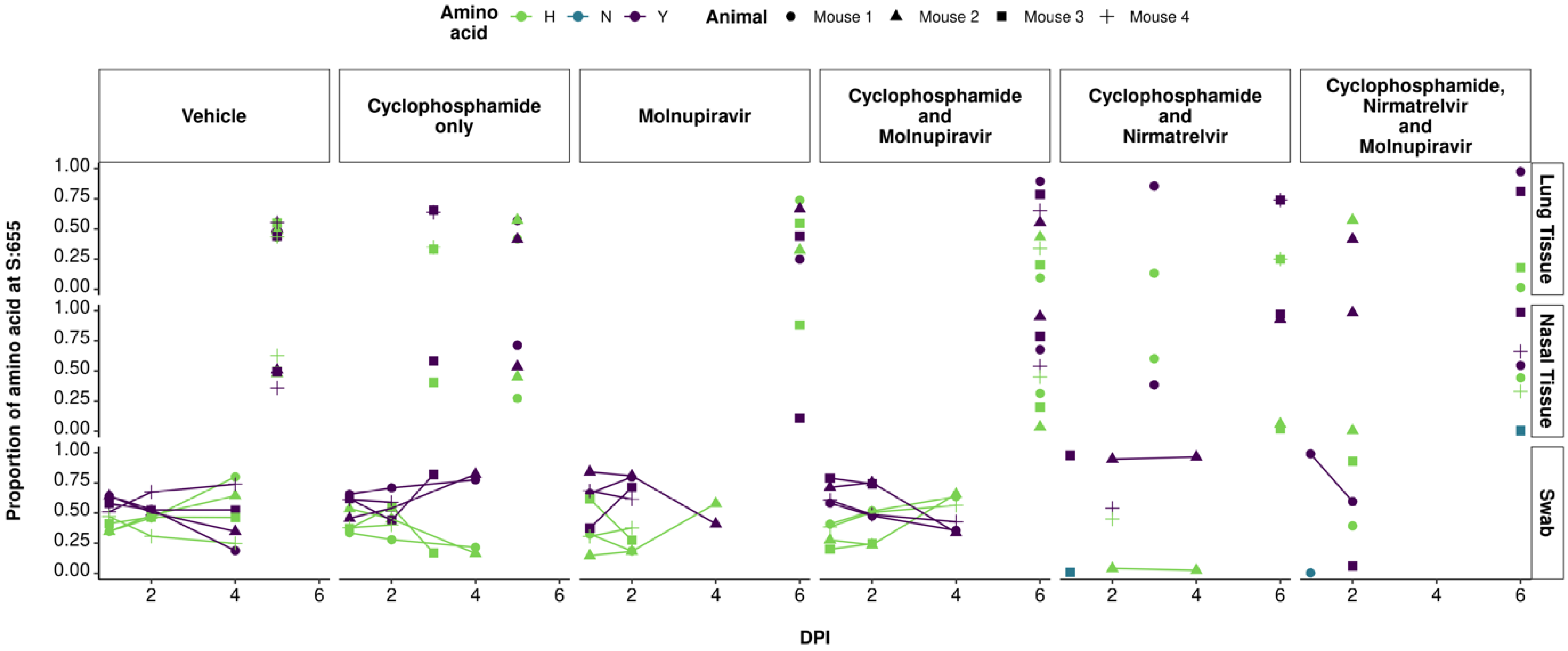

The proportion of amino acid H and Y at position 655 in the Spike protein over time or in lung or nasal tissues. The S: H655Y mutation was observed at the minor variant level if not observed in the consensus level. Each point represents one animal within the cohort.
